# MiR-410 Is Overexpressed in Liver and Colorectal Tumors and Enhances Tumor Cell Growth by Silencing FHL1 via a Direct/Indirect Mechanism

**DOI:** 10.1371/journal.pone.0108708

**Published:** 2014-10-01

**Authors:** Yu Wang, Jie Fu, Mengmeng Jiang, Xiaoai Zhang, Long Cheng, Xiaojie Xu, Zhongyi Fan, Jing Zhang, Qinong Ye, Haifeng Song

**Affiliations:** 1 Department of Pharmacology and Toxicology, Beijing Institute of Radiation Medicine, Beijing, China; 2 SDU School of Life Science, Jinan, China; 3 State Key Laboratory of Pathogen and Biosecurity, Beijing Institute of Microbiology and Epidemiology, Beijing, China; 4 Department of Medical Molecular Biology, Beijing Institute of Biotechnology, Beijing, China; H. Lee Moffitt Cancer Center & Research Institute, United States of America

## Abstract

FHL1 is an important tumor-suppressor that is downregulated in multiple tumors by unknown mechanisms. We demonstrated that miR-410 specifically targets the 3′UTR of FHL1. Furthermore, using DNA bisulfite modification and sequencing experiments, we demonstrated that the FHL1 promoter is hypermethylated in cancer cells. FHL1 methylation is increased upon miR-410 expression, suggesting that the regulation of FHL1 by miR-410 occurs by a dual mechanism. Using chromatin immunoprecipitation assays, we observed that miR-410 overexpression results in the increased binding of DNMT3A at the FHL1 promoter, which could explain how miR-410 regulates FHL1 methylation. Importantly, *in vitro* and *in vivo* results suggest that miR-410 may have oncogenic properties. Furthermore, both miR-410 and DNMT3A are upregulated in clinical human liver and colorectal tumors cancers. Our results suggest that miR-410 may function as an oncomiR and are consistent with its key function in regulating FHL1 in certain digestive system cancers.

## Introduction

The inactivation of tumor-suppressor genes is a characteristic step in cancer development and progression [1]. Among the mechanisms of tumor-suppressor gene inactivation, aberrant DNA methylation of gene promoter islands and endogenous miRNA upregulation lead to the silencing of tumor-suppressor genes in multiple tissues [2], [3].

The four-and-a-half LIM (FHL) proteins are a family of LIM-only proteins that regulate cell proliferation, differentiation, and apoptosis. Studies using clinical samples have shown that FHL1 expression is down-regulated in multiple human tumor types, including gastric cancer and hepatocarcinoma. FHL1 exerts tumor suppressor function via multiple mechanisms, including the activation of the TGF-β-like and Src-MAPK signaling pathways and protein interaction with ZO-1, HIF1α, and ERα [4]–[8]. Although the role of FHL1 in cancer development and progression is well established, the detailed mechanisms of how FHL1 is down-regulated during carcinogenesis remain unknown.

MicroRNAs (miRNAs) are a class of short, highly conserved, non-coding RNAs that function as negative posttranscriptional regulators of target genes [9], [10], [11]. Accumulating evidence has shown that miRNAs are aberrantly expressed during the development and/or progression of a variety of human cancers [12], [13]. Roles for miRNAs in the regulation of tumorigenesis via the targeting of important genes within signaling pathways are evident [14], [15], [16]. To date, however, miRNAs that target FHL1 have not been reported.

To clarify the mechanism of FHL1 downregulation during tumorogenesis, we assessed the involvement of miRNAs that target the FHL1 promoter. We report that miR-410 is upregulated in colorectal cancer and hepatocarcinoma and that miR-410 can decrease FHL1 protein levels both directly by targeting the FHL1 3'UTR and indirectly by promoting the up-regulation of DNA methylases. In particular, miR-410 promotes the binding of DNMT3A to the FHL1 promoter, which leads to the hypermethylation of the FHL1 promoter. The findings here could provide a mechanism for FHL1 down-regulation during tumorigenesis. Consistently, analysis of clinical hepatocarcinoma and colorectal specimens revealed a physiological association between the expression of miR-410, FHL1 and DNMT3A, which implicates miR-410 as a potential oncogenic biomarker that functions by down-regulating FHL1 in these tissues.

## Materials and Methods

### Ethics statement

This study was performed with the approval of the Ethical Committee of the Beijing Institute of Radiation Medicine and conducted according to the principles expressed in the Declaration of Helsinki. Written informed consent was obtained from all the participants before inclusion in the study. All procedures involving animals were approved by the Institute of Animal Care and Use Committee at the Academy of Military Medical Sciences (AMMS). The animal study was carried out in strict accordance with the recommendations in the Guide for the Care and Use of Laboratory Animals of the Beijing Institute of Radiation Medicine.

### Plasmids and miRNA antagomir

miRNA precursors were a gift from Professor Xiaofei Zheng. The miRNA precursor sequences were cloned into pcDNA3.0 vector (Invitrogen, Carlsbad, CA) for use in transient transfection. Hsa-miR-410 antagomir was chemically synthesized and purified by high-performance liquid chromatography (Gene Pharma, Shanghai, China) for use in transient transfection. The miR-410 precursor sequences were also cloned into pCDH-CMV-MCS-EF1-Puro vector (System Biosciences, North Whisman, CA) to construct pCDH-miR-410 for preparation of stable miRNA-expressing HepG2 cells used in animal experiments.

The wt-FHL1 3′UTR-Luc reporter was created by ligation of a FHL1 3′UTR PCR product into a Luciferase-modified pcDNA3.0 vector. The 3′UTR of the human FHL1 gene was obtained by PCR using the following primers: 5′-CCGGAATTCACTGACAGGGGCTCCTGTCC-3′ (forward) and 5′-CCGCTCGAGCATATGCTGCTTTATTTCTG-3′ (reverse). A mutant reporter, Mut-FHL1-3′UTR, was generated from wt-FHL1-3′UTR by mutating the target site within the region predicted to be recognized by the seed region of miR410. The wild-type seed recognition sequence located at 1154–1159 bp of the FHL1 3′UTR (TTATAT) was mutated to a mutant seed recognition sequence (CCACAC). For this purpose, two pairs of primers were designed to obtain the two separate PCR fragments. For one fragment (1168 bp), the primers were as follows: 5′-ACTGACAGGGGCTCCTGTCC-3′ (410 FHL1 forward) and 5′-TACTATGTCGTGTGGTAATGTAGA-3′ (410 mut FHL1 reverse). For the other fragment (179 bp), the primers were as follows: 5′-TCTACATTACCACACGACATAGTA-3′ (410 mut FHL1 forward) and 5′-CATATGCTGCTTTATTTCTG-3′ (410 FHL1 reverse). Overlapping PCR was then performed using the two recovered fragments as templates and 410 FHL1 forward and 410 FHL1 reverse as primers to obtain the Mut-FHL1-3′UTR PCR fragment. The mut-FHL1 3′UTR-Luc reporter was created by ligation of the product into the Luciferase-modified pcDNA3.0 vector.

### Cell culture and transfection

Human HepG2 and LS180, and 293T cells were maintained in our laboratory. HepG2 and 293T cells were purchased from ATCC [17], [18] and the LS180 was a gift from Professor Lu [19]. HepG2/siFHL1, a HepG2 cell line stable expressing siRNA against FHL1, and its matched control cell, HepG2siCTRL, were constructed as previously described [8]. Cells were maintained in Dulbecco’s modified Eagle’s medium (Gibco, Los Angeles, CA, USA) supplemented with 10% fetal bovine serum (Gibco), and were incubated at 37°C in a 5% CO_2_ atmosphere. Cell transfection was performed using NanoFectin (System Biosciences) according to the manufacturer’s instructions. Stable HepG2 or LS180 cell lines overexpressing miR-410 were generated by lentiviral transduction using pCDH-miR-410 according to the manufacturer's instructions.

### Luciferase reporter assays

Cells were cultured in 24-well plates and then transfected with wt-FHL1-3'UTR or Mut-FHL1-3′UTR together with miRNA precursor-expressing plasmid or empty vector control. A vector expressing Renilla luciferase, pRL-TK (Promega, Southampton, UK) was cotransfected for normalization. After 48h incubation, luciferase activity was measured using a dual-luciferase reporter system (Promega). All transfection experiments were performed in triplicate and reproduced at least 3 times.

### Cell proliferation and migration assays

Anchorage-dependent cell growth was assessed using a CCK-8 Kit (Dojindo Laboratories, Mashikimachi, Japan) according to the manufacturer’s instructions for HepG2/control, HepG2/miR-410, and HepG2/miR-410 antagomir (HepG2/miR-410 transfected with miR-410 antagomir) cell lines. The absorbance values of each well were measured with a microplate spectrophotometer (Molecular Devices, Sunnyvale, CA) at 450 nm. All proliferation assays were repeated as independent experiments at least 3 times.

For transwell migration assays, HepG2/control, HepG2/miR-410, and HepG2/miR-410 antagomir cells (10^5^ cells each) were plated onto the upper chamber of an 8 µm pore Transwell filter (Corning Costar, Tewksbury, MA). The upper chamber was placed in DMEM containing 1% serum and the lower chamber 10% serum. After 24 hours, unmigrated cells were removed from the upper chamber with a cotton swab. The remaining cells that had migrated through the membrane were fixed, stained with 0.1% crystal violet and photographed under a microscope. The cells were then extracted with 10% acetic acid for absorbance determination at 570 nm.

### Real-time reverse-transcription-PCR (RT-PCR) and Western blotting

Total RNA from cells and tissues was isolated using TRIzol Reagent (Invitrogen) and reverse transcribed using Revert Aid First Strand cDNA Synthesis Kit (Thermo Scientific, Waltham, MA). RT-PCR was performed with the primers listed in Table S1 using Maxima SYBR Green/Fluorescein qPCR Master Mix (Thermo Scientific). Gene expression levels were normalized to GAPDH, and the fold change of target genes was calculated using the 2^−ΔΔCt^ method.

Immunoblotting was performed using antibodies against FHL1 (1:1000 dilution, Proteintech, Chicago, USA), and GAPDH (1:1000 dilution, Santa Cruz, Dallas, USA). Experiments were repeated at least 3 times.

### miRNA extraction and qRT-PCR

Total RNA from tissues or cell lines was extracted using miRNeasy Mini kits (Qiagen, Crawley, UK). Target miRNAs were reverse transcribed to cDNA using gene-specific RT primers with the miRCURY LNA™ Universal RT microRNA PCR/Universal cDNA Synthesis Kit II (Exiqon, Woburn, MA, USA). miRNA expression profiles of tissues or cell lines were determined using the miScript SYBR Green PCR Kit (Qiagen) with the ABI7000 Real-Time PCR System (Applied Biosystems, Carlsbad, CA, USA). Primers for miRNA quantification were miRCURY LNA Universal primer (hsa-miR-410, hsa-miR-214, and hsa-miR-495) and U6 snRNA PCR primer (Exiqon). The relative quantification value of the target, normalized to the U6 snRNA control, was calculated by the comparative Ct method.

### Bisulfite sequencing

Genomic DNA was isolated from Human Umbilical Vein Endothelial Cells (HUVEC), HepG2/control cells, or HepG2/miR-410 cells. Bisulfite modification was performed with the EpiTect Bisulfite Kit (Qiagen) according to the manufacturer’s recommendations. The −27 to +181 bp FHL1 promoter sequence (http://genome.ucsc.edu/genome), which contains 10 theoretical CpG sites, was amplified from bisulfite-treated DNA using the following primers: BSP-sense: 5'-AAGTTTTTAGGGTAGGGTG-3'; BSP-antisense: 5'-AAAACTAAATAAACCCC-.

ATCCATAAT-3'. The PCR products were cloned into pGEM-T Easy (Promega), and 10 positive clones were sequenced for each specimen.

### Chromatin Immunoprecipitation (ChIP) Assay

ChIP of genomic DNA associated with a methyl FHL1 was carried out according to the manufacturer’s protocol (Millipore, Billerica, MA, USA). miR-410 overexpression and control HepG2 and LS180 cells were cross-linked in formaldehyde, and then the reactions were stopped with the addition of glycine. After washing twice in ice-cold PBS containing a protease inhibitor cocktail, cell lysates were harvested in SDS lysis buffer and sonicated to produce about 200∼1000 bp genomic fragments, which were verified by electrophoresis in 2% DNA agarose. The supernatant was used for immunoprecipitation at 4°C overnight with 5 µg of mouse polyclonal DNMT3A antibody (Abcam, Cambridge, UK) or anti-mouse IgG (Vector Laboratories, Burlingame, CA, USA) as a negative control. A portion of the sonicated DNA was left untreated to serve as input control. Immune complexes were collected on protein G beads and washed according to the manufacturer’s protocol. Immunoprecipitated DNA was subjected to quantitative real-time PCR (qPCR) using GAPDH primers from the EZ-ChIP Kit and ChIP primers amplifying a specific region (−920 bp∼807 bp) of the FHL1 promoter (5'-ACCGAGTGAGAAAAGCCAAT-3'/5'-TCACCATTGGCAACCACTGAT-3'). After each ChIP assay, the input and IP samples were subjected to qPCR, and FHL1 signals were normalized to GAPDH to determine whether the immunoprecipitate was enriched. QPCR protocol and reaction conditions were as recommended by the EZ-ChIP Kit. The PCR products were isolated by agarose gel electrophoresis and visualized by ethidium bromide staining using a Gel Documentation 2000 system (Bio-Rad, Hercules, CA).

### Animal experiments

For *in vivo* tumor growth assays, HepG2 cells stably transfected with pCDH or pCDH-miR-410 were injected subcutaneously into the hind legs of 4-week-old male nude mice (n = 8). Tumor size was measured at the indicated times using calipers. Tumor volume was calculated according to the following formula: volume = (longest diameter × shortest diameter^2^)/2. *In vivo* experiments with LS180 cells were similar to those with HepG2 cells.

### FHL1, DNMT3A, and miR-410 expression analysis in liver and colorectal tumors

We harvested 20 human colorectal and 33 liver tumors and their matched paracancerous tissues for analysis of the association between FHL1, DNMT3A, and miR-410 expression using qRT-PCR. DNMT3A was assessed as an indirect indicator of the FHL1 methylation status.

### Statistics

Differences between variables were assessed by *χ* ^2^ analysis, using the 2-tailed Student’s *t* test. *P* values of less than 0.05 were considered statistically significant.

## Results

### miR-410 targets the 3′ UTR of FHL1

To investigate candidate miRNAs that regulate FHL1 expression in cancer, we used 2 target prediction programs, TargetScan and miRanda. Our analysis predicted 17 potential FHL1-targeting miRNAs. To assess the ability of these miRNAs to target the FHL1 3′UTR, we cloned the 3′-UTR sequence of human FHL1 into the luciferase expression vector pcDNA 3.0-luc. Preliminary screening results showed that miR-146a, miR-146b-5p, miR-214, miR-495, and miR-410 can significantly inhibit luciferase activity (Figure. S1A). To verify the specificity of inhibition, we constructed a mutated vector Mut-FHL1-3'UTR, which harbors a mutation in the targets that are predicted to be recognized by the miRNA seed regions (Figure. S1B). We transiently expressed a control or miRNA expression plasmid together with the wt-FHL1 3'UTR or Mut-FHL1 3'UTR plasmids. Results verify that each of the 5 miRNAs could inhibit the activity for the wt-FHL1 3'UTR luciferase plasmid; however, mutation of the seed recognition sequence prevented a decrease in luciferase activity only for miR-410, miR-495 and miR-214 (Figure. S1C). These data suggest that miR-410, miR-495, and miR-214 specifically inhibit the FHL1 3′UTR, but that the effects of miR-146a and miR-146b-5p may be non-specific or at sites other than their predicted target sites.

To further investigate the ability of these 5 miRNAs to suppress FHL1 expression, we assessed the levels of FHL1 protein in miRNA-expressing 293T cells. Western blot analysis showed that miR-410, miR-495, and miR-214 could inhibit FHL1 protein expression the most dramatically (Figure. S2A). Interestingly, FHL1 mRNA expression also was decreased by miR-410, but not miR-495 or miR-214 (Figure. S2B). These results suggest that miR-495 and miR-214 inhibit FHL1 at the protein level, but that miR-410 functions to inhibit miR-410 via a mechanism that occurs prior to FHL1 translation.

### miR-410 promotes FHL1 methylation

Recent evidence suggests that some miRNAs can regulate gene expression indirectly by epigenetic effects on methylation, rather than direct effects on RNA degradation or translation [14], [15], [16]. To explore the possibility that miR-410 may regulate the methylation status of the FHL1 gene, we prepared HepG2 liver cells and LS180 colon carcinoma cells over-expressing either miR-410 or control oligonucleotides. The levels of the mRNAs for the DNA methylases DNMT1, DNMT3A, and DNMT3B were upregulated in both the HepG2/miR-410 and LS180/miR-410 cells, with the most dramatic upregulation observed for DNMT3A (Figure. S3A). To determine whether the expression of these methylases correlated with the effects on FHL1 promoter methylation, the DNA methylation status of the FHL1 promoter region was analyzed by bisulfate sequencing using primers designed to cover a region from the −27 bp upstream transcription start site to 181 bp downstream (Figure. S3B). We focused on the ten CpG islands (CGIs) within the FHL1 promoter as an indicator of methylation status. As shown in Figure. S3C, the CGIs of HUVECs, as a normal control cell, were mostly unmethylated, whereas HepG2/control liver cancer cells were mostly methylated. Notably, when miR-410 was overexpressed in HepG2/miR-410 cells, FHL1 was completely methylated. These results suggest that methylation occurs in HepG2 tumor cells and that the expression of miR-410 induces further methylation. Because promoter hypermethylation is associated with gene silencing, the effects of miR-410 on FHL1 promoter methylation could provide a basis for the silencing of FHL1 mRNA expression by miR-410.

### miR-410 promotes DNMT3A binding to the promoter of FHL1

ChIP experiments were performed to analyses the recruitment of DNMT3A to the FHL1 promoter. A total of 5 different primer pairs spanning −3000 bp ∼ +1 bp with about 500 bp intervals were designed to determine the critical promoter region of FHL1 for accumulating DNMT3A. Results showed that DNMT3A accumulated at about −920 bp ∼807 bp. Consistent with the bisulfite sequencing results, ChIP analysis demonstrated that miR-410 increased the FHL1 promoter-associated DNMT3A binding by 3- to 4 -fold in HepG2 cells (Figure. S4) and LS180 cells (Diagram S1A). Taken together, the correlation of increased FHL1 promoter-associated CpG island hypermethylation with increased DNMT3A binding and decreased FHL1 expression supports the idea that DNA methylation in the FHL1 promoter region controls its expression.

### miR-410 releases FHL1 suppression of carcinoma cell growth both in vitro and in vivo

To assess the tumor-enhancer potential of miR-410, we compared the growth properties of control miRNA and miR-410 in HepG2 and LS180 cells. As an additional specificity control, we also tested HepG2/miR-410 cells transfected with miR-410 antagomir (HepG2/miR-410 antagomir cells). The HepG2/miR-410 cells were verified to have increased miR-410 and decreased FHL1 levels, which was reversed by concomitant expression of the antagomir (Figure. S5A, top 2 panels). CCK-8 assays showed that miR-410 overexpression led to increased proliferation compared with the control, and that the phenotype could be rescued by co-transfection of miR-410 antagomir (Figure. S5A, bottom panel). To verify the oncogenic properties conferred by miR-410 expression, transwell assay assays showed that the migration ability of HepG2 cells with miR-410 overexpression was enhanced and that miR-410 antagomir reversed this effect (Figure. S4B). Similar observations were determined in LS180 cell lines (Diagram S1B,1C).

To determine whether the biological function of miR-410 is primarily attributed to FHL1 knockdown, we assessed the effects of miR-410 plasmid or control oligonucleotides transfected into HepG2 cells that express either a specific siRNA against FHL1 (siFHL1) or a control siRNA (siCTRL). As shown in Figure. S4C, siFHL1 dramatically promoted proliferation as compared to siCTRL. In fact, the efficacy of the siRNA appeared to be greater than the miRNA, potentially because siRNAs typically have increased specificity in knocking down expression, while miR-410 is predicted to regulate other genes besides FHL1 that control cell growth. Nevertheless, these results verify that miR-410 has similar physiological effects to siFHL1 in promoting HepG2 cell growth.

To determine whether the observed *in vitro* phenotype of miR-410 overexpression also affects *in vivo* tumor growth, we examined the growth of HepG2 cell lines in nude mice. Consistent with an *in vivo* oncogenic role for miR-410, the tumors in mice inoculated with HepG2/miR-410 cells or LS180/miR-410 cells grew significantly faster than the tumors in mice inoculated with HepG2/control cells (Figure. S5D) and LS180/control cells (Diagram S1D). Collectively, these data indicate that miR-410 introduction suppresses FHL1 expression and promotes tumor survival both *in vitro* and *in vivo*.

### Association between FHL1, DNMT3A, and miR-410 expression in vivo and in clinical samples

To determine whether the association between FHL1, miR-410, and DNMT3A expression has physiological relevance to human cancer, the expression of these three genes was analyzed in tumors harvested from HepG2 and LS180-injected mice and clinical samples (20 human colorectal and 33 liver tumors and their paracancerous tissues). As shown in Figure. S6A and Diagram S1E, the *in vivo* expression pattern of FHL1, miR-410, and DNMT3A was consistent with the *in vitro* results. Furthermore, of the 20 colorectal clinical samples, 15/20 (75%) showed decreased FHL1 expression and 14/20 (70%) showed elevated miR-410 expression as compared to their matched normal tissues (*P*<0.001). DNMT3A expression was increased in 12/20 (60%) of the colorectal tumors (*P*<0.05). Similar results were obtained for the 33 liver tumors, with 27/33 (82%) showing decreased FHL1 expression and 25/33 (76%) showing elevated miR-410 expression as compared to their matched normal tissues (*P*<0.001). DNMT3A expression was increased in 20/33 liver tumors (61%) (*P*<0.05). As the colorectal and liver samples are derived from the digest tract tumor, we combined the data in the Figure S6. The expression of FHL1 in the carcinoma tissues was less on average than the expression in the paracancerous (normal) tissues (*P* = 0.009, n = 55). Consistent with its role as a negative regulator of FHL1, miR-410 expression was dramatically elevated in tumors as compared to their matched normal tissues (*P* = 0.0001, n = 55). Furthermore, DNMT3A expression was increased (*P* = 0.045, n = 55). These results reveal a potential mechanism by which miR-410 overexpression in carcinomas leads to the down-regulation of FHL1 expression by directly targeting of the FHL1 3′UTR and also by promoting the expression of DNA methylases, such as DNMT3A, which additionally repress FHL1 mRNA expression through DNA hypermethylation.

## Discussion

The findings here within indicate, for the first time, that miR-410 may serve as a regulator of FHL1 expression through direct targeting of its 3'UTR and indirect regulation of its methylation in human colorectal cancer and hepatocarcinoma. Recent reports have shown other possible functions of miR-410. miR-410 is involved in the regulation of lipoprotein lipase levels, muscle regeneration, and idiopathic pulmonary fibrosis [20], [21], [22]. Also, miR-410 is considered a diagnostic and prognostic biomarker in some diseases. In a study based on plasma samples from 122 women (77 endometrioid endometrial cancer and 45 controls), tumor tissues with a combination of miR-92a/miR-410 or miR-92a/miR-205/miR-410 expression was shown to serve as a noninvasive biomarker for early cancer detection and prognosis [23]. Therefore, there is a precedent for a possible role of miR-410 as a biomarker for malignant diseases. Our results also show that expression is elevated in clinical tissue from colorectal and liver tumors; however, based on our limited sample size (20 human colorectal and 33 liver tumors), the use of a larger sample size may be required to confirm the potential utility of miR-410 as a biomarker for colorectal and liver tumors.

FHL1 is a well known tumor-suppressor gene with no or relatively low expression in tumors. Methylation-silenced mutation is one of the important mechanisms contributing its low-expression [24]. Here, we have shown another new mechanism for silencing of FHL1 expression in tumors: miR-410 not only specifically targets the 3′UTR of FHL1, but also promotes DNMT3A binding to the FHL1 promoter, which leads to its hypermethylation in cancer cells, suggesting that the regulation of FHL1 by miR-410 occurs by dual mechanisms. Exogenous miR-410 expression leads to the upregulation of methylases, and both miR-410 and DNMT3A are upregulated in human colorectal and liver tumors cancers. These results are consistent with other studies in which the down-regulation of FHL1 in multiple carcinomas was demonstrated to be caused primarily by methylation [24], [25]. Regulation of methylation also provides an explanation for the miR-410-mediated decrease in FHL1 both at the level of mRNA and protein, whereas two other miRNAs (miR-214, and miR-495) down-regulate FHL1 expression only at the protein level. The ability of miR-410 to upregulate DNMTs is in contrast to results indicating that other miRNAs, including miR-29b, down-regulate DNMT3A and DNMT3B by targeting their 3' UTRs or down-regulate DNMT1 indirectly by targeting the 3'UTR of Sp1, a transactivator of the DNMT1 gene [14], [15], [16]. Indeed, it is also possible that miR-410 may target DNMT3A indirectly through down-regulation of an inhibitory transcriptional factor or a miRNA that targets DNMT3A. This possibility was not suggested by bioinformatic analysis of miR-410 targets (not shown), and thus, additional studies may distinguish between these possible mechanisms of miR-410-mediated effects on FHL1 promoter methylation.

Previous evidence also shows that CpG islands in the upstream regions of 18 pre-miRNAs are methylated after 5-azacytidine treatment, including miR-663, miR-369, miR-615 and miR-410. However, only miR-663 could be regulated by DNA methylation, and the expression levels of miR-369, miR-615, and miR-410 were not regulated by DNA methylation in K562 cells [26]. Nevertheless, the down-regulation of miR-410 expression in neuroblastoma and gliomas might be related to its methylation status [27], [28]. Therefore, it is possible that miR-410 may have a role as both a target and effector of gene methylation, suggesting that miR-410 expression may be under the control of an autoregulatory loop that involves its ability to enhance DNMT3 levels.

Importantly, our results also show that miR-410 may have oncogenic properties. These results were shown in vitro using both proliferation and migration assays, as well as in vivo by injection of miR-410-expressing HepG2 cells into nude mice. Our results suggest that miR-410 may function as an oncomiR and are consistent with a key regulatory function for miR-410 upregulation in regulating FHL1 in colorectal and liver tumors cancers. Further studies may verify the classification of miR-410 as an oncomiR in colorectal and liver tumors and also validate its potential utility as a biomarker.

## Supporting Information

Figure S1 **Interaction between miRNAs and the 3′-UTR of FHL1.** (A) Dual luciferase assay of 293T cells cotransfected with firefly luciferase constructs containing the FHL1 and pre-miRNAs as indicated. The firefly luciferase activity was normalized to Renilla luciferase activity. The data are shown as relative luciferase activity of pre-miRNAs transfected cells with respect to the control (scrambled oligonucleotide). Experiments were repeated at least 3 times. Data are shown as mean ± sd. (B) Schema of the four firefly luciferase reporter constructs for FHL1 3′UTR, indicating the predicted interaction sites between miR-146a/146b-5p, miR-410, miR-495, miR-214 and the FHL1 3′UTR. Mut-FHL1-3′UTRs were constructed by mutating the seed recognition sequences. (C) Dual luciferase assay of 293T cells cotransfected with firefly luciferase constructs containing a wild-type or mutated FHL1 3′UTR and pre-miRNA or scrambled oligonucleotides. Experiments were repeated at least 3 times. Data were shown as mean ± sd. * *P*<0.05, wt vs mut.
(TIF)

Figure S2 **miRNAs down-regulate FHL1 expression.** (A) Immunoblot analysis of the endogenous FHL1 protein expression in 293T cells after transient transfection with pre-miRNAs or scramble oligonucleotides controls. Equivalent gel loading was confirmed using internal GAPDH. (B) Real-time RT-PCR analysis of FHL1 mRNA in HepG2 cells stably transfected with pre-miR-410, pre-miR-495, pre-miR-214 or scrambled oligonucleotide control. Histograms show fold changes (reduction) in mRNA expression with respect to the control after normalization with GAPDH. Data shown are mean ± sd of triplicate measurements that were repeated 3 times with similar results. ** *P*<0.01 versus corresponding controls.
(TIF)

Figure S3 **miR-410 promotes DNA methylase expression and FHL1 promoter methylation** ***in vitro*** **.** (A) Real-time RT-PCR analysis of the DNA methylases, DNMT3A, DNMT3B, and DNMT1 in HepG2 or LS180 cells stably transfected with pre-miR-410 or scrambled oligonucleotide control. Histograms show fold changes in mRNA expression with respect to the control after normalization with GAPDH. Data shown are mean ± sd of triplicate measurements that were repeated 3 times with similar results. *P<0.05, ** *P*<0.01, *** *P*<0.01 versus corresponding controls. (B) Genomic structure of FHL1 and a CpG map of its promoter CpG Islands (CGI). Arrow, transcription start site (TSS); vertical lines, individual CpG sites. (C) Confirmation of FHL1 methylation status by bisulfite sequencing of normal HUVECs, HepG2 cancer cells, and HepG2 cells stably transfected with miR-410. In total, ten CpG sites were analyzed. HUVECs were designated as the normal control. Closed circle, methylated CpG site; open circle, unmethylated CpG site.
(TIF)

Figure S4 **miR410 promotes DNMT3A binding to the FHL1 promoter as assessed by ChIP assay.** pCDH vector-control (Control) and miR-410 expression vector (miR-410) were transfected into HepG2 cells, which were subjected to ChIP assay using anti-DNMT3A or anti-mouse IgG antibody (IgG) and amplified by qPCR. (A). The immunoprecipitates from the ChIP assay were subjected to qPCR with FHL1 primer and normalized to GAPDH. *P<0.05 compared with the Control group. Each experiment was repeated three times. Data are expressed as mean ± sd. (B). PCR products were detected by agarose gel electrophoresis. 1) H_2_O-template negative control; 2) Anti-DNMT3A IP in HepG2/miR-410 cells;3) Anti-DNMT3A IP in HepG2/control cells;4) IgG IP in HepG2/miR-410 cells;5) IgG IP in HepG2/control cells;6–8) The same with the 1–5) except that the templates were input.
(TIF)

Figure S5 **miR-410 promotes HepG2 growth** ***in vitro*** **and** ***in vivo*** **in a FHL1-dependent manner.** (A) HepG2 cells were stably transfected with miR-410 or control oligonucleotides, and then were transfected with miR-410 antagomir. miR-410 was quantified by RT-QPCR and FHL1 was quantified by immunoblotting to verify the three cell lines (top 2 panels). Cell growth assays of the three cell lines were analyzed (bottom panel). Data shown are mean ± sd of triplicate measurements. * *P*<0.05 versus control on day 3. ** *P*<0.01 versus control on day 4. (B) Cell viability was assessed using migration transwell assays at the indicated times. Scale bar, 100 µm. *P<0.05 versus control. (C) Cell growth assays were analyzed. SIFHL1-control: HepG2 cells were transfected stably with FHL1 siRNA and transiently with control oligonucleotide; siCTRL-miR-410: HepG2 cells were transfected stably with Scramble siRNA and transiently with miR-410; siCTRL-control: HepG2 cells were transfected stably with Scramble siRNA and transiently with control oligonucleotide; SiFHL1-miR-410: HepG2 cells were transfected stably with FHL1 siRNA and transiently with miR-410. * *P*<0.05 versus siCTRL-control. **P<0.01 versus siCTRL-control. (D) Volume of xenograft tumors derived from HepG2 cells expressing control oligonucleotides or miR-410. Data are shown as mean ± sd (n = 8). ** *P*<0.01 versus control. *** *P*<0.001 versus control.
(TIF)

Figure S6 **Expression of FHL1, miR-410 and DNMT3A in mouse tumors and surgical human liver and colorectal cancer specimens.** (A) FHL1, miR-410 and DNMT3A expression level were determined in HepG2 tumors harvested from *in vivo* animal experiments (*n* = 3). * *P*<0.05 versus control. ** *P*<0.01 versus control. (B) Decreased expression of FHL1, and increased expression of miR-410 and DNMT3A in digest tract tumors (colorectal and liver cancers) (*n* = 55) compared with the paracancerous tissues analyzed by qRT–PCR. A horizontal line represents the mean expression level in each group. *P<0.05; **P<0.01; ***P<0.01.
(TIF)

Table S1 **Primers used for real-time RT-PCR.**
(DOC)

Diagram S1 **miR-410 promotes tumor growth** ***in vitro*** **and** ***in vivo*** **by downregulating FHL1** ***via*** **methylation regulation in LS180 cells.** (A) LS180 cells were transiently transfected with miR410 or control oligonucleotides and then were subjected to ChIP assay as detailed in the legend to Fig. S4. Results are shown for QPCR (top panel) and PCR product determination by agarose gel electrophoresis (bottom panel). (B) LS180 cells transiently transfected with miR-410 or control oligonucleotides. miR-410 was quantified by RT-QPCR and FHL1 expression was quantified by immunoblotting to verify the cell lines (left 2 panels). Cell growth assays were analyzed using validated cell lines (right panel). Data shown are mean ± sd of triplicate measurements. * *P*<0.05 versus control. (C) Cell viability was assessed using migration transwell assays 48 h post transfection. Scale bar, 100 µm. *P<0.05 versus control. (D) Volume of xenograft tumors derived from LS180 cells expressing control oligonucleotides or miR-410. Data are shown as mean ± sd (*n* = 8). * *P*<0.05, ** *P*<0.01 versus control. (E) RT-qPCR analysis of DNMT3A, FHL1 and miR-410 in LS180 tumors harvested from *in vivo* animal experiments. * *P*<0.05 versus control.
(TIF)
