# Supplementary figures and images for: MiR-410 Is Overexpressed in Liver and Colorectal Tumors and Enhances Tumor Cell Growth by Silencing FHL1 via a Direct/Indirect Mechanism
Source: PLoS One. 2014 Oct 1;9(10):e108708. doi: 10.1371/journal.pone.0108708 (PMC4182719; doi:10.1371/journal.pone.0108708)

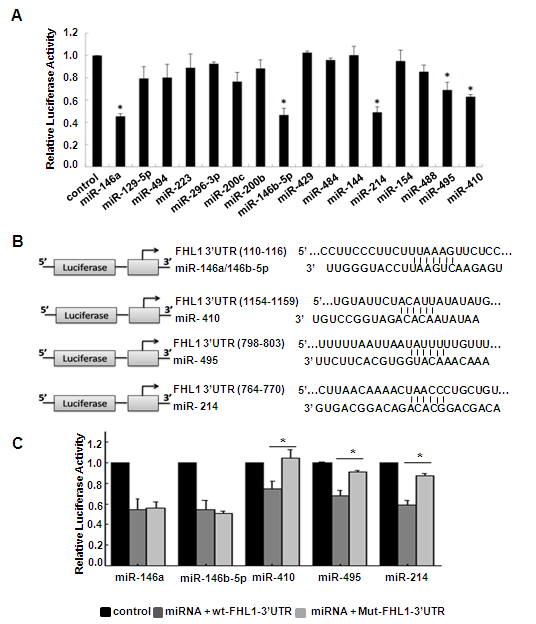

Supplement: Figure S1 — Interaction between miRNAs and the 3′-UTR of FHL1. (A) Dual luciferase assay of 293T cells cotransfected with firefly luciferase constructs containing the FHL1 and pre-miRNAs as indicated. The firefly luciferase activity was normalized to Renilla luciferase activity. The data are shown as relative luciferase activity of pre-miRNAs transfected cells with respect to the control (scrambled oligonucleotide). Experiments were repeated at least 3 times. Data are shown as mean ± sd. (B) Schema of the four firefly luciferase reporter constructs for FHL1 3′UTR, indicating the predicted interaction sites between miR-146a/146b-5p, miR-410, miR-495, miR-214 and the FHL1 3′UTR. Mut-FHL1-3′UTRs were constructed by mutating the seed recognition sequences. (C) Dual luciferase assay of 293T cells cotransfected with firefly luciferase constructs containing a wild-type or mutated FHL1 3′UTR and pre-miRNA or scrambled oligonucleotides. Experiments were repeated at least 3 times. Data were shown as mean ± sd. * P<0.05, wt vs mut. (TIF) [file pone.0108708.s001.tif]

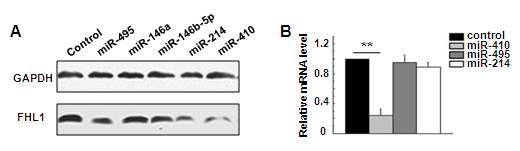

Supplement: Figure S2 — miRNAs down-regulate FHL1 expression. (A) Immunoblot analysis of the endogenous FHL1 protein expression in 293T cells after transient transfection with pre-miRNAs or scramble oligonucleotides controls. Equivalent gel loading was confirmed using internal GAPDH. (B) Real-time RT-PCR analysis of FHL1 mRNA in HepG2 cells stably transfected with pre-miR-410, pre-miR-495, pre-miR-214 or scrambled oligonucleotide control. Histograms show fold changes (reduction) in mRNA expression with respect to the control after normalization with GAPDH. Data shown are mean ± sd of triplicate measurements that were repeated 3 times with similar results. ** P<0.01 versus corresponding controls. (TIF) [file pone.0108708.s002.tif]

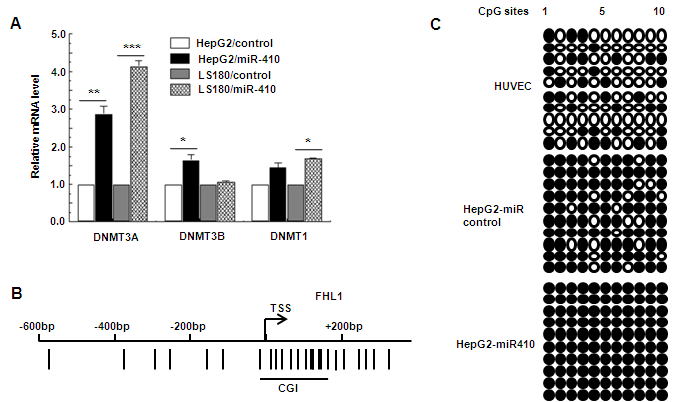

Supplement: Figure S3 — miR-410 promotes DNA methylase expression and FHL1 promoter methylation in vitro . (A) Real-time RT-PCR analysis of the DNA methylases, DNMT3A, DNMT3B, and DNMT1 in HepG2 or LS180 cells stably transfected with pre-miR-410 or scrambled oligonucleotide control. Histograms show fold changes in mRNA expression with respect to the control after normalization with GAPDH. Data shown are mean ± sd of triplicate measurements that were repeated 3 times with similar results. *P<0.05, ** P<0.01, *** P<0.01 versus corresponding controls. (B) Genomic structure of FHL1 and a CpG map of its promoter CpG Islands (CGI). Arrow, transcription start site (TSS); vertical lines, individual CpG sites. (C) Confirmation of FHL1 methylation status by bisulfite sequencing of normal HUVECs, HepG2 cancer cells, and HepG2 cells stably transfected with miR-410. In total, ten CpG sites were analyzed. HUVECs were designated as the normal control. Closed circle, methylated CpG site; open circle, unmethylated CpG site. (TIF) [file pone.0108708.s003.tif]

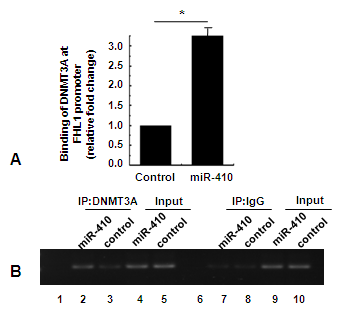

Supplement: Figure S4 — miR410 promotes DNMT3A binding to the FHL1 promoter as assessed by ChIP assay. pCDH vector-control (Control) and miR-410 expression vector (miR-410) were transfected into HepG2 cells, which were subjected to ChIP assay using anti-DNMT3A or anti-mouse IgG antibody (IgG) and amplified by qPCR. (A). The immunoprecipitates from the ChIP assay were subjected to qPCR with FHL1 primer and normalized to GAPDH. *P<0.05 compared with the Control group. Each experiment was repeated three times. Data are expressed as mean ± sd. (B). PCR products were detected by agarose gel electrophoresis. 1) H2O-template negative control; 2) Anti-DNMT3A IP in HepG2/miR-410 cells;3) Anti-DNMT3A IP in HepG2/control cells;4) IgG IP in HepG2/miR-410 cells;5) IgG IP in HepG2/control cells;6–8) The same with the 1–5) except that the templates were input. (TIF) [file pone.0108708.s004.tif]

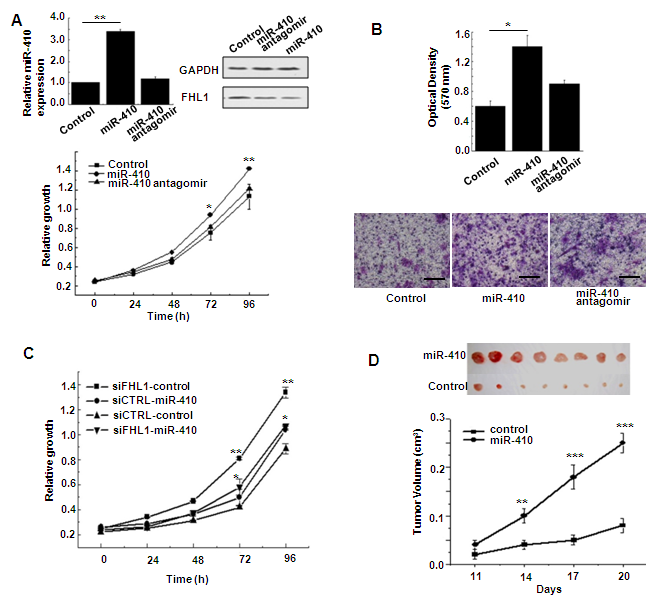

Supplement: Figure S5 — miR-410 promotes HepG2 growth in vitro and in vivo in a FHL1-dependent manner. (A) HepG2 cells were stably transfected with miR-410 or control oligonucleotides, and then were transfected with miR-410 antagomir. miR-410 was quantified by RT-QPCR and FHL1 was quantified by immunoblotting to verify the three cell lines (top 2 panels). Cell growth assays of the three cell lines were analyzed (bottom panel). Data shown are mean ± sd of triplicate measurements. * P<0.05 versus control on day 3. ** P<0.01 versus control on day 4. (B) Cell viability was assessed using migration transwell assays at the indicated times. Scale bar, 100 µm. *P<0.05 versus control. (C) Cell growth assays were analyzed. SIFHL1-control: HepG2 cells were transfected stably with FHL1 siRNA and transiently with control oligonucleotide; siCTRL-miR-410: HepG2 cells were transfected stably with Scramble siRNA and transiently with miR-410; siCTRL-control: HepG2 cells were transfected stably with Scramble siRNA and transiently with control oligonucleotide; SiFHL1-miR-410: HepG2 cells were transfected stably with FHL1 siRNA and transiently with miR-410. * P<0.05 versus siCTRL-control. **P<0.01 versus siCTRL-control. (D) Volume of xenograft tumors derived from HepG2 cells expressing control oligonucleotides or miR-410. Data are shown as mean ± sd (n = 8). ** P<0.01 versus control. *** P<0.001 versus control. (TIF) [file pone.0108708.s005.tif]

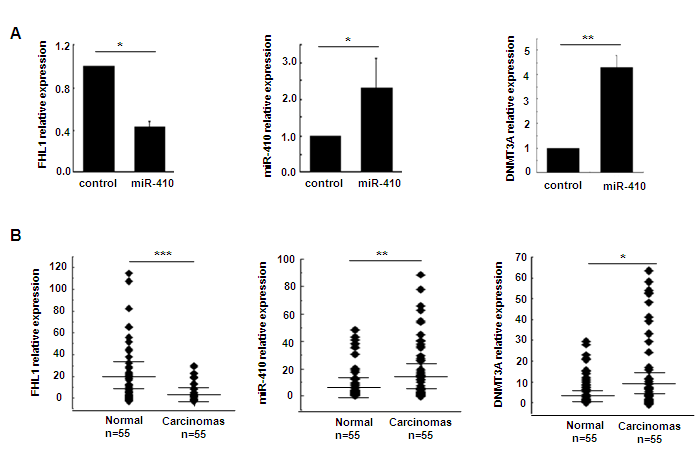

Supplement: Figure S6 — Expression of FHL1, miR-410 and DNMT3A in mouse tumors and surgical human liver and colorectal cancer specimens. (A) FHL1, miR-410 and DNMT3A expression level were determined in HepG2 tumors harvested from in vivo animal experiments (n = 3). * P<0.05 versus control. ** P<0.01 versus control. (B) Decreased expression of FHL1, and increased expression of miR-410 and DNMT3A in digest tract tumors (colorectal and liver cancers) (n = 55) compared with the paracancerous tissues analyzed by qRT–PCR. A horizontal line represents the mean expression level in each group. *P<0.05; **P<0.01; ***P<0.01. (TIF) [file pone.0108708.s006.tif]

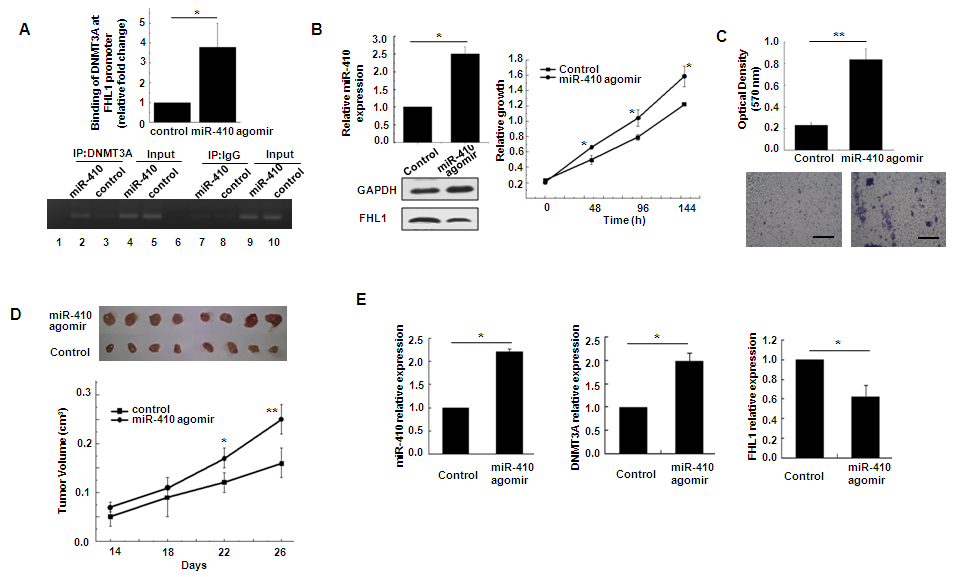

Supplement: Diagram S1 — miR-410 promotes tumor growth in vitro and in vivo by downregulating FHL1 via methylation regulation in LS180 cells. (A) LS180 cells were transiently transfected with miR410 or control oligonucleotides and then were subjected to ChIP assay as detailed in the legend to Fig. S4. Results are shown for QPCR (top panel) and PCR product determination by agarose gel electrophoresis (bottom panel). (B) LS180 cells transiently transfected with miR-410 or control oligonucleotides. miR-410 was quantified by RT-QPCR and FHL1 expression was quantified by immunoblotting to verify the cell lines (left 2 panels). Cell growth assays were analyzed using validated cell lines (right panel). Data shown are mean ± sd of triplicate measurements. * P<0.05 versus control. (C) Cell viability was assessed using migration transwell assays 48 h post transfection. Scale bar, 100 µm. *P<0.05 versus control. (D) Volume of xenograft tumors derived from LS180 cells expressing control oligonucleotides or miR-410. Data are shown as mean ± sd (n = 8). * P<0.05, ** P<0.01 versus control. (E) RT-qPCR analysis of DNMT3A, FHL1 and miR-410 in LS180 tumors harvested from in vivo animal experiments. * P<0.05 versus control. (TIF) [file pone.0108708.s008.tif]
