# Supplementary material for: MiR-410 Is Overexpressed in Liver and Colorectal Tumors and Enhances Tumor Cell Growth by Silencing FHL1 via a Direct/Indirect Mechanism
Source: PLoS One. 2014 Oct 1;9(10):e108708. doi: 10.1371/journal.pone.0108708 (PMC4182719; doi:10.1371/journal.pone.0108708)
Supplement: Table S1 — Primers used for real-time RT-PCR. (DOC) [file pone.0108708.s007.doc]

Table S1. Primers used for real-time RT-PCR.

| Gene | Forward (5'→3') | Reverse (5'→3') |
| --- | --- | --- |
| FHL1 | CTGCTGCCTGAAA | TCTCCTGCCACAAT |
| DNMT1 | GGGGACCCACGAAA | ACCTCACAGACGCCAC |
| DNMT3A | TACCCACCTGTCCCA | GCGGTCCACCTGAAT |
| DNMT3B | AGTTGGGCATAAAGG | TGGCTGGATTCACATT |
| GAPDH | GAAGGTGAAGGTCGGAGTC | GAAGATGGTGATGGGATTTC |
